# Is incentive spirometry beneficial for patients with lung cancer receiving video-assisted thoracic surgery?

**DOI:** 10.1186/s12890-019-0885-8

**Published:** 2019-07-08

**Authors:** Chin-Jung Liu, Wen-Chen Tsai, Chia-Chen Chu, Chih-Hsin Muo, Wei-Sheng Chung

**Affiliations:** 1 Department of Health Services Administration, China Medical University, Taichung, Taiwan; 2 Department of Public Health, China Medical University, Taichung, Taiwan; 3 Department of Respiratory Therapy, China Medical University, Taichung, Taiwan; 4 Division of Respiratory Therapy, China Medical University Hospital, Taichung, Taiwan; 5 Department of Biomedical Engineering, Chung Yuan Christian University, Jhongli, Taiwan; 6 Management Office for Health Data, China Medical University Hospital, Taichung, Taiwan; 7 Department of Internal Medicine, Taichung Hospital, Ministry of Health and Welfare, No. 199, Section 1, San-Min Road, Taichung City, 40343 Taiwan; 8 Department of Healthcare Administration, Central Taiwan University of Science and Technology, Taichung, Taiwan

**Keywords:** Lung cancer, Incentive spirometry, Video-assisted thoracic surgery (VATS)

## Abstract

**Background:**

The effectiveness of Incentive spirometry (IS) in patients undergoing video-assisted thoracic surgery (VATS) remains lacking. We conducted a population-based study to investigate the effectiveness of IS on patients with lung cancers following VATS.

**Methods:**

We identified patients newly diagnosed with lung cancer who underwent surgical resection by VATS or thoracotomy from the years 2000 to 2008 in the Longitudinal Health Insurance Database. Exposure variable was the use of IS during admission for surgical resection by VATS or thoracotomy. Primary outcomes included hospitalization cost, incidence of pneumonia, and length of hospital stay. Secondary outcomes included the frequency of emergency department (ED) visits and hospitalizations at 3-month, 6-month, and 12-month follow-ups after thoracic surgery.

**Results:**

We analyzed 7549 patients with lung cancer undergoing surgical resection by VATS and thoracotomy. The proportion of patients who were subjected to IS was significantly higher in those who underwent thoracotomy than in those who underwent VATS (68.4% vs. 53.1%, *P* < 0.0001). After we controlled for potential covariates, the IS group significantly reduced hospitalization costs (− 524.5 USD, 95% confidence interval [CI] = − 982.6 USD – -66.4 USD) and the risk of pneumonia (odds ratio = 0.55, 95% CI = 0.32–0.95) compared to the non-IS group following VATS. No difference in ED visit frequency and hospitalization frequency at 3-month, 6-month, and 1-year follow-up was noted between the IS and the non-IS groups following VATS.

**Conclusions:**

The use of IS in patients with lung cancers undergoing VATS may reduce hospitalization cost and the risk of pneumonia.

## Background

Lung cancer is one of the leading causes of cancer-related deaths in the world [1]. Non-small cell lung cancers (NSCLCs) are the most common types of lung cancers and have diverse pathological characteristics [2]. Survival from lung cancer is highly associated with lung resection surgery [3]. Surgery of NSCLCs includes wedge resection, sleeve lobectomy, lobectomy, and pneumonectomy if there is no contraindication [4–6]. Thoracotomy and video-assisted thoracic surgery (VATS) are common approaches for managing stage I and II NSCLCs undergoing surgical resection with lymph node dissection or sampling [6–8]. VATS, which provides a minimally invasive approach, may reduce the incidence of pulmonary complications and improve survival rates compared with thoracotomy [9–11].

The most common postoperative complications following thoracic or abdominal surgery are pulmonary complications, such as atelectasis (alveolar collapse), pneumonia, and acute respiratory failure [12, 13]. Lung expansion therapy allows patients to maintain an effective cough mechanism to facilitate removal of secretions from the airways following surgery. An incentive spirometer is a medical device, which helps patients sustain maximal inspiration under visual quantitation by inspiratory effort. Incentive spirometry (IS) has become the anchor of lung expansion therapy, which provides an effective strategy to restore preoperative pulmonary function for surgical patients [14].

Although physiological evidence suggests that IS may be effective for lung re-expansion after surgery, studies are controversial about the degree of benefit [15, 16]. Cochrane systematic reviews did not show evidence of IS benefit in lowering postoperative pulmonary complications and improving pulmonary dynamics in patients receiving coronary bypass grafting and upper abdominal surgery [15, 17]. However, the size and methodology of these studies were of low quality. The American Association for Respiratory Care (AARC) Clinical Practice Guidelines recommended that IS be used with deep breathing techniques, directed coughing, early mobilization, and optimal analgesia to prevent postoperative pulmonary complications [14]. The IS may measure and assist lung expansion in order to prevent pulmonary complications following thoracic surgery [18]. Some clinicians have widely considered IS as a part of perioperative respiratory strategies to prevent complications. In Taiwan, a major discrepancy existed on the physicians’ belief for clinical effectiveness of the IS use. Studies on the effectiveness of IS on patients undergoing surgical resection of lung cancer via VATS remain lacking. Thus, we conducted a retrospective study using population-based data to investigate the effectiveness of IS on patients with lung cancers undergoing surgical resection via VATS or thoracotomy.

## Methods

### Data sources

The government implemented the mandatory National Health Insurance (NHI) program in 1995, which covers 99% of the residents in Taiwan [19]. We analyzed the data for this study from the Longitudinal Health Insurance Database for Catastrophic Illness Patients (LHID-CIP) of the National Health Insurance Research Database (NHIRD) established by the National Health Insurance Administration (NHIA, formerly Bureau of National Health Insurance). The data in the LHID-CIP were derived from a sub-data set of the NHIRD that comprised one million randomly sampled beneficiaries enrolled in the NHI program in 2012 and contained all the records on these insurants from 1996 to 2012. Personal identification in NHIRD was recorded before release to researchers. LHID-CIP included all medical claims of each catastrophic illness patient, who suffered from malignancy, autoimmune disorders, and other catastrophic diseases. For validation, the catastrophic illness is based on the clinical report and review of 2 specialists under the NHIA guideline [19]. Diseases were defined in NHIRD according to the International Classification of Diseases, Ninth Revision, Clinical Modification (ICD-9-CM). This study was approved by the institutional review board of China Medical University in central Taiwan (CMUH104-REC2–115). Previous studies have indicated a high accuracy of the ICD-9-CM diagnoses in the NHIRD [20, 21].

### Study patients

**Fig. 1:**
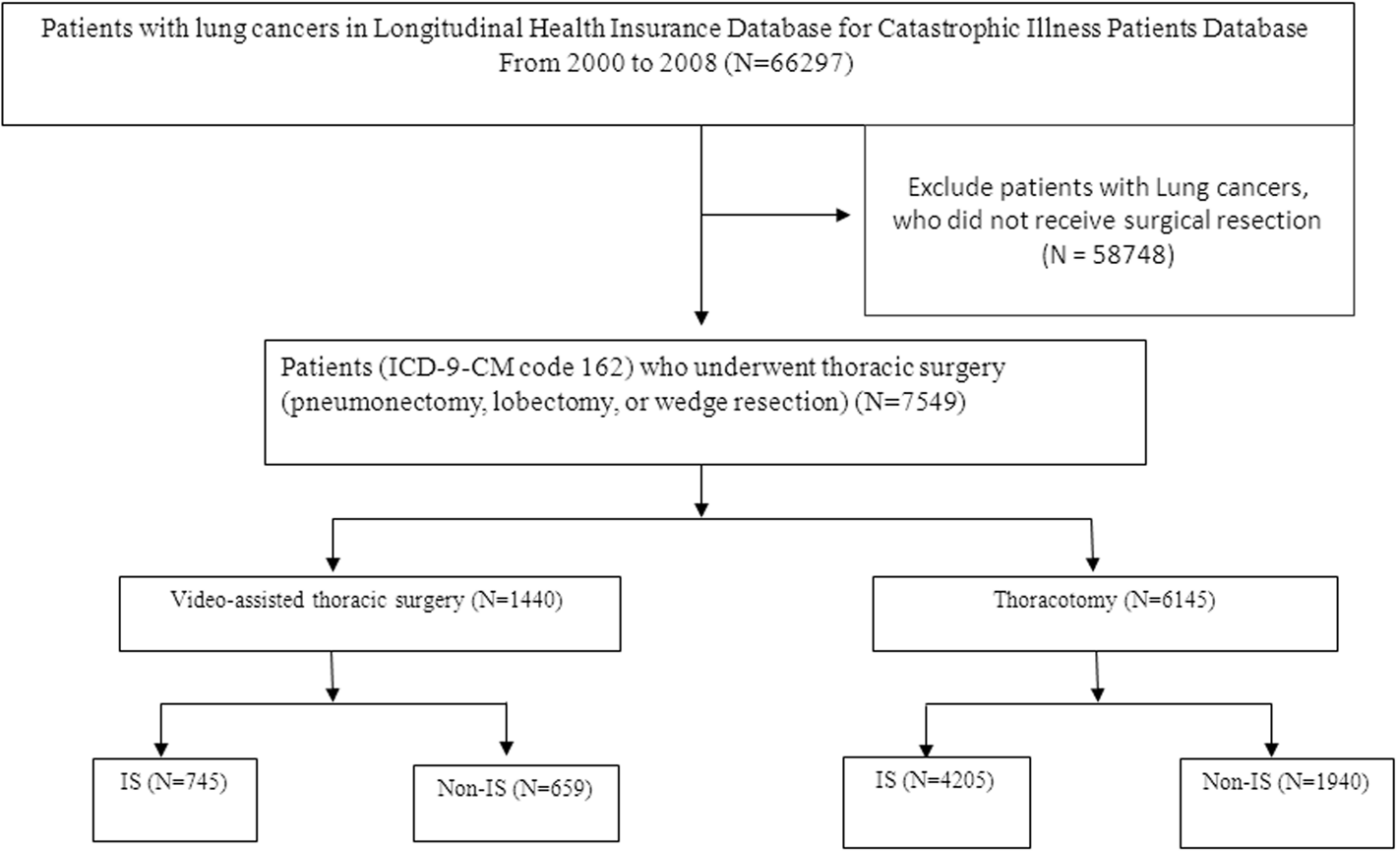
Flow diagram of the study patients

We identified 66,297 patients with a new diagnosis of lung cancer (ICD-9-CM code 162) in the LHID-CIP from 2000 to 2008. Inclusion criteria included patients newly diagnosed with lung cancers who underwent surgical resection by VATS or thoracotomy. Index date was defined as the date of surgical resection by thoracotomy or VATS. We excluded 58,748 patients with lung cancers, who did not receive surgical resection (Fig. 1).

### Exposure measures

AARC Clinical Practice Guidelines did not comment on the use of IS in patients following thoracic surgery. The chest surgeons encouraged early mobility and ambulation for patients following thoracic surgery in Taiwan. In addition, some of the chest surgeons prescribed IS for patients with lung cancers receiving surgical resection via VATS or thoracotomy, although this is outside the clinical guidelines. We defined exposure variable as the use of IS (procedure code 57011B) within 7 days of admission for surgical resection by VATS or thoracotomy due to Health insurance payment limited within 7 days after surgery [22] and IS showed a good correlation with vital capacity (VC) in the first 8 days after surgery [23]. The Taiwan National Health Insurance Administration (NHIA) requested the respiratory therapist to conduct IS and reimbursed by the procedure code 57011B. Therefore, the respiratory therapists have to instruct the patient to use IS and follow up the performance [22].The respiratory therapist instructed the IS use in accordance with AARC clinical practice guideline incentive spirometry and recorded in the medical chart for administrative review [14, 24]. The NHIA supervises the medical records routinely by administrative and peer review. The respiratory therapist instructed patients to take slow and deep breaths using the spirometer and hold their breaths for a minimum of 3 s. Patients performed incentive spirometer with 5 to 10 sustained maximal inspiratory maneuvers each hour.

### Outcome measures

We evaluated the effect of IS in patients admitted with lung cancer receiving surgical resection by VATS or thoracotomy. Primary outcomes included hospitalization cost, incidence of pneumonia (ICD-9-CM codes 481–486), acute respiratory distress syndrome (ARDS, ICD-9-CM code 518.82), and length of stay during the surgical admission. Furthermore, the patient owned the device of IS when they trained to use the IS. The patients were instructed to train the IS in addition to deep breathing and cough training after discharge. Therefore, Secondary outcomes included the frequency of emergency department (ED) visits and the frequency of hospitalizations during serial follow-up periods. The follow-up periods were classified as short-term (3-m), intermediate-term (6-m), and long-term (12-m).

### Covariates

We categorized age into 4 groups: < 55 years, 55–64 years, 65–74 years, and > 75 years. Comorbidities included chronic obstructive pulmonary disease (COPD, ICD-9-CM codes 491, 492, 496) and asthma (ICD-9-CM code 493), heart failure (ICD-9-CM code 428), and other cancer (ICD-9-CM codes 140–208 except 162). In addition, we also collected neoadjuvant therapy (radiation and chemotherapy) and the extent of surgery including pneumonectomy (procedure code 32.5), lobectomy (procedure codes 32.3 and 32.4), and limited resection (procedure code 32.29).

### Statistical analyses

Distribution of demographics (including sex and age groups) and comorbidities (including COPD, asthma, and other cancer) between IS and non-IS groups in patients with lung cancers undergoing VATS or thoracotomy were compared using Chi-square test. Mean ages were measured and compared using student’s t-test. Multivariate linear regression was used to estimate hospitalization cost, length of stay, ED visit frequency, and hospitalization frequency between IS and non-IS groups after controlling for potential covariates. Multivariate logistic regression was used to evaluate the risk of pneumonia and ARDS between IS and non-IS groups after controlling for potential covariates and presented with odds ratios (ORs) and 95% confidence intervals (CIs). Statistical analysis was performed using SAS version 9.4 (SAS Institute Inc., Cary, NC, USA). The statistical significance level was set at 0.05.

## Results

A total of 7549 patients (4478 men and 3071 women) with lung cancers underwent surgical resection. Mean age was 63.2 ± 11.3 years. Among these patients, the medical comorbid disorders exhibited were COPD (57.5%), asthma (15.4%), heart failure (4.42%), and other cancer (8.4%). Most of the patients (81.4%) with lung cancers received surgical resection via thoracotomy. The chest surgeons prescribed IS for 65.5% of patients with lung cancers undergoing surgical resection. 66.3% of the patients received neoadjuvant. Most of the patients (92.1%) received lobectomy (Table 1).

**Table 1:** Demographic characteristics and comorbidities of patients with lung cancers receiving surgical resection

Variables	*N* = 7549	%
Sex		
Women	3071	40.7
Men	4478	59.3
Mean age, year (SD)	63.2	11.3
< 55	1834	24.3
55–64	2051	27.2
65–74	2587	34.3
75+	1077	14.3
Comorbidity		
COPD	4340	57.5
Asthma	1160	15.4
Other cancer	636	8.4
Heart failure	334	4.42
Approach		
VATS	1404	18.6
Thoracotomy	6145	81.4
Incentive spirometry		
Yes	4950	65.6
No	2599	34.4
Neoadjuvant		
Yes	5004	66.3
No	2545	33.7
Procedure		
Pneumonectomy	11	0.15
Lobectomy	6951	92.1
Limited resection	587	7.78

*VATS* video-assisted thoracic surgery

A total of 1404 patients with lung cancers underwent surgical resection by VATS (VATS cohort) while 6145 patients underwent thoracotomy (thoracotomy cohort). The distributions of sex and age were similar between the IS group and the non-IS group in both cohorts. No significant difference was noted in the mean age of the patients. The IS group had a lower prevalence of asthma and a higher proportion of neoadjuvant than the non-IS group in the thoracotomy cohort (*P* < 0.05) (Table 2).

**Table 2:** Comparison of demographics and comorbidities of patients with lung cancers receiving surgical resection by VATS and thoracotomy

	VATS *N* = 1404					Thoracotomy *N* = 6145				
	IS *N* = 745 (53.1%)		Non-IS *N* = 659 (46.9%)			IS *N* = 4205 (68.4%)		Non-IS *N* = 1940 (31.6%)		
	n	%	n	%	*p*-value	n	%	n	%	p-value
Sex					0.44					0.35
Female	384	51.5	326	49.5		1599	38.0	762	39.3	
Male	361	48.5	333	50.5		2606	62.0	1178	60.7	
Mean age, year (SD)	63.1	(11.7)	62.3	(11.7)	0.16	63.2	(11.1)	63.4	(11.3)	0.63
< 55	207	27.8	167	25.3	0.52	999	23.8	461	23.8	0.96
55–64	206	27.7	181	27.5		1147	27.3	517	26.7	
65–74	235	31.5	209	31.7		1462	34.8	681	35.1	
75+	97	13.0	102	15.5		597	14.2	281	14.5	
Comorbidity										
COPD	389	52.2	365	55.4	0.23	2435	57.9	1151	59.3	0.29
Asthma	136	18.3	102	15.5	0.17	604	14.4	318	16.4	0.04
Other cancer	96	12.9	79	12.0	0.61	313	7.44	148	7.63	0.80
Heart failure	45	6.04	40	6.04	0.98	166	3.95	83	4.28	0.54
Neoadjuvant	453	60.8	386	58.6	0.39	2896	68.9	1269	65.4	0.007
Procedure method					0.58					0.66
Pneumonectomy	5	0.67	2	0.30		3	0.07	1	0.05	
Lobectomy	428	57.5	388	58.9		4199	99.9	1936	99.8	
Limited resection	312	41.9	269	40.8		3	0.07	3	0.15	

Chi-square test, Fisher’s exact test and Student t test; *IS* incentive spirometry

**Fig. 2:**
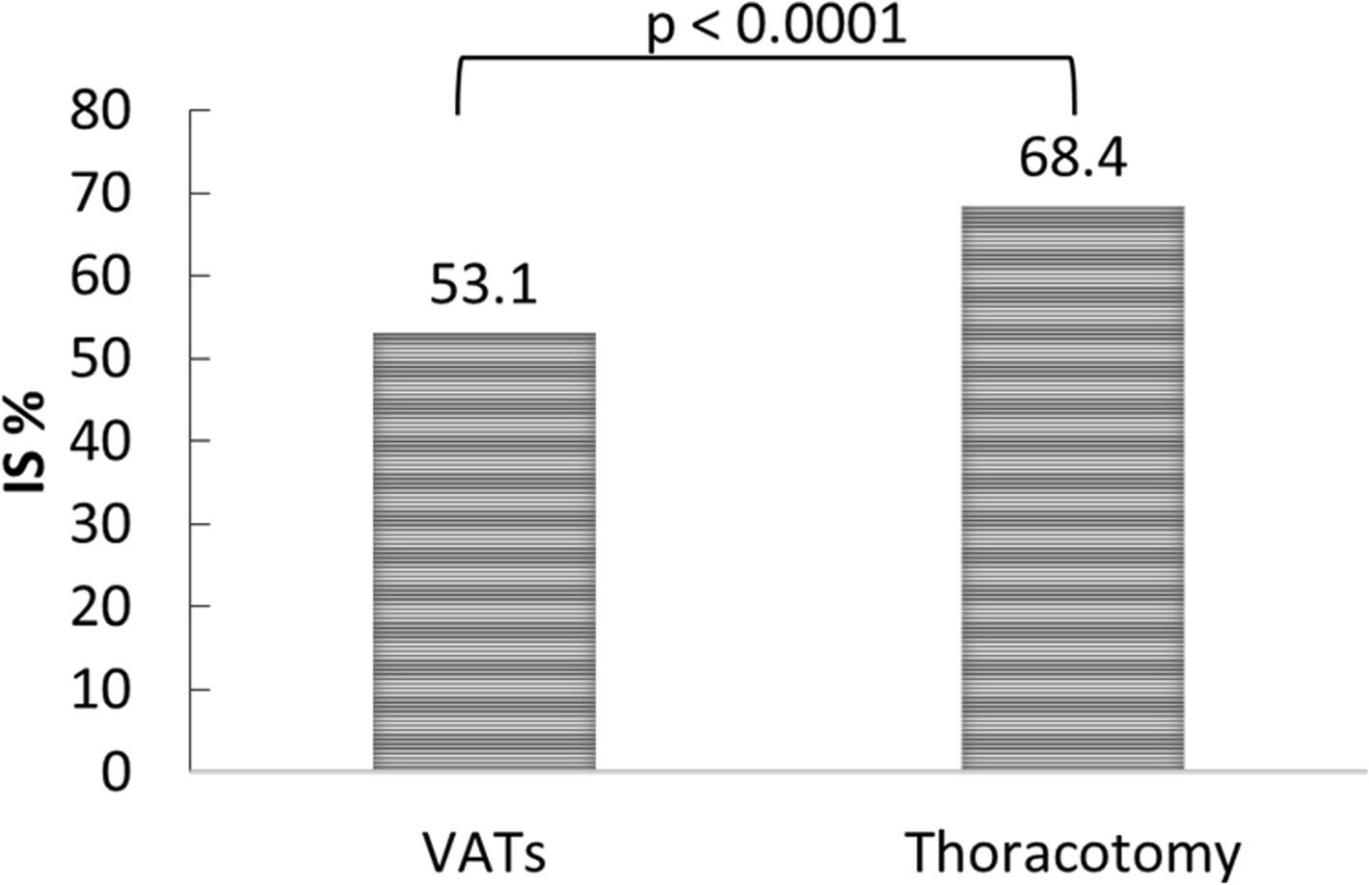
Proportion of IS use between patients undergoing VATS and thoracotomy

The proportion of IS use in patients who underwent thoracotomy for lung cancer was significantly higher than in those who underwent VATS (68.4% vs. 53.1%, *P* < 0.0001) (Fig. 2).

The IS group exhibited lower median hospitalization costs than the non-IS group, regardless of whether VATS (5295.5 USD vs. 5536.5 USD) or thoracotomy (4864.6 USD vs. 4904.6 USD) was used. After we controlled for potential covariates, the IS group still exhibited significantly lower hospitalization costs than the non-IS group, regardless of whether VATS or thoracotomy. The proportion of pneumonia development was less in the IS group than in the non-IS group following VATS (3.09% vs. 5.46%) and thoracotomy (5.56% vs. 6.19%). However, the IS group showed significantly lower risk of pneumonia than the non-IS group in patients receiving VATS after controlling for potential covariates (OR = 0.55, 95% CI = 0.32–0.95). No difference in ED visit frequency and hospitalization frequency existed between the 2 groups, regardless of VATS or thoracotomy used during serial follow-up periods (Table 3).

**Table 3:** Comparison of outcome between IS and non-IS groups in patients with lung cancers undergoing surgical resection via VATs and thoracotomy

	VATS								Thoracotomy							
	IS			Non-IS			IS vs. Non-IS		IS			Non-IS			IS vs. Non-IS	
Outcome	Mean	SD	Median	Mean	SD	Median	Estimate	(95% CI)	Mean	SD	Median	Mean	SD	Median	Estimate	(95% CI)
Hospitalization cost (USD)^1^	5976.8	3823	5295.5	6568.4	4994.5	5536.5	−524.5	(−982.6, −66.4)*	5668.3	4851.4	4864.6	6425.9	7440.6	4904.6	− 705.5	(− 1013, − 398.3)***
Length of stay (d)^1^	19.3	80.9	13	18.3	40.1	13	1.13	(−5.72, 7.97)	19.5	20.4	16	23.3	117.6	17	−3.60	(−7.27, 0.07)
Pneumonia (n, %)^2^	23	3.09		36	5.46		0.55	(0.32, 0.95)*	234	5.56		120	6.19		0.90	(0.72, 1.14)
ADRS (n, %)^2^	0	0.00		2	0.30		NA		3	0.07		2	0.10		0.82	(0.14, 4.97)
3-month outcome																
ED visit frequency^1^	0.27	0.69	0	0.28	0.93	0	−0.02	(−0.10, 0.07)	0.30	0.78	0	0.31	0.85	0	−0.02	(−0.06, 0.03)
Hospitalization frequency^1^	0.59	1.06	0	0.63	1.08	0	−0.05	(−0.16, 0.05)	0.54	1.01	0	0.50	0.98	0	0.02	(−0.03, 0.07)
6-month outcome																
ED visit frequency^1^	0.48	1.02	0	0.51	1.31	0	− 0.03	(− 0.15, 0.09)	0.54	1.22	0	0.55	1.23	0	−0.01	(− 0.08, 0.05)
Hospitalization frequency^1^	1.20	1.89	0	1.24	2.01	0	−0.07	(− 0.26, 0.11)	1.16	1.91	0	1.05	1.74	0	0.08	(−0.02, 0.18)
1-year outcome																
ED visit frequency^1^	0.79	1.50	0	0.94	2.24	0	−0.17	(−0.36, 0.03)	0.98	1.99	0	0.93	1.66	0	0.04	(−0.06, 0.14)
Hospitalization frequency^1^	1.81	2.72	0	1.83	2.80	0	−0.08	(−0.34, 0.18)	1.87	2.77	0	1.74	2.64	0	0.07	(−0.07, 0.21)

1 USD = 31.54 NTD in 2008; IS: incentive spirometry;

^1^Linear regression after adjusted for age, gender, COPD, asthma, other cancer, heart failure, neoadjuvant and procedure method

^2^Logistic regression after adjusted for age, gender, COPD, asthma, other cancer, heart failure, neoadjuvant and procedure method

* *p* < 0.05, ** *p* < 0.01, *** *p* < 0.001

## Discussion

The IS is a simple way to measure the inspiratory vital capacity (VC) at bedside, and has a good correlation with VC after lung surgery [23]. We investigated the effectiveness of IS in patients with lung cancers receiving surgical resection by VATS in Taiwan by using a population-based study. Our study demonstrated lower hospitalization costs (− 524.5 USD, 95% CI = − 982.6 USD – -66.4 USD) and decreased risk of pneumonia (OR = 0.55, 95% = 0.32–0.95) in the IS group than in the non-IS group for patients receiving surgical resection by VATS.

Failure to clear airway secretions remains to be a major concern for patients undergoing thoracic surgery. Although manual percussion with chest wall vibration is widely applied in respiratory physiotherapy, it is labor-intensive and time-consuming. Moreover, postoperative pain may interfere with performance of percussion [25]. The purpose of incentive spirometer is for patients to take a sustained maximal inspiratory effort, which allows a large inflating volume and transpulmonary pressure gradient for several seconds to reach lung expansion [18]. The use of IS assists the maintenance of airway patency at risk of closure and removes retained airway secretions [26]. In addition, IS provides patients with visual feedback in terms of volume, with regard to inspiratory effort to reach a measurable goal and encourage adherence to therapy [14]. However, no statistical difference of pneumonia risk was present between IS and non-IS groups for patients receiving surgical resection by open thoracotomy. More than 99% of the patients in open thoracotomy received major lung resection in the current study. The reason may be related to major lung function loss and postoperative pain [16].

The literatures did not recommend to routinely use IS for prevention of postoperative collapse [14, 15, 17, 27–29]. The current study showed 65.5% of patients with lung cancers to use the IS undergoing surgical resection. The chest physicians and respiratory therapists encouraged patients to deep breathing, cough, and early ambulation whether the use of IS or not. Early ambulation and other cofounders may influence these long-term outcomes [30–32]. The current study showed no significant difference of pneumonia risk between IS and non-IS groups for patients with lung cancer undergoing lung resection by thoracotomy. However, the IS group significantly reduced the risk of pneumonia (odds ratio = 0.55, 95% CI = 0.32–0.95) compared to the non-IS group for the patients following VATS. Thus, randomized controlled trial of IS in patients undergoing lung resection by VATS may be warranted in the future study.

In our study, we found the mean hospitalization costs by VATS is higher than by thoracotomy, irrespective of IS use (5977 USD in VATS vs. 5668 USD in thoracotomy) or non-use (6568 USD in VATS vs. 6425 USD in thoracotomy). Reimbursement for medical costs in the NHI program is higher for VATS than for thoracotomy (1018 USD vs. 812 USD) [19]. However, VATS may reduce operation time, duration of tube thoracostomy and hospital days, and mortality rate [10, 33]. Compared with conventional open thoracotomy, VATS gives the same 5-year overall survival rates for NSCLCs [34]. Recently, less invasive procedures are becoming more popular, such as laparoscopic surgery and VATS, to minimize the surgical wound. Studies have reported that patients undergoing VATS exhibited decreased painkiller use, lower complication rates, shorter hospital length of stay, better lung function reserve, and higher scores on the quality of life scale than those who underwent open thoracotomy did [11, 35–41].

The hospitalization cost was lower in the IS group than that in the non-IS group regardless of receiving open thoractomy or VATS. Compared to the non-IS group, the IS group showed significantly lower risk of pneumonia for the patients undergoing VATS, which may decrease hospitalization cost. No significant difference of pneumonia was present between the two groups for patients undergoing thoracotomy. However, The finding of lower hospiatlization cost in the IS group was compatible with the study by the Torrington & Henderson [42]. We recommend the need for further clinical observational studies.

Our study showed that LOS for the the patients with lung cancer undergoing VATS or open thoracotomy was 18.3–19.3 days and 19.5–23.3 days between 2000 and 2008. The findings were consistent with previous studies using NHIRD from 2004 to 2010 [43, 44]. The LOS of the current study was longer than a study by Flores et al. [45], which may be associated with various reimbursement system, pay for service in Taiwan NHI and diagnosis related group payment system in the USA.

The use of population-based data is our strength, since it is highly representative of the general population. The NHI program is universal and mandatory in Taiwan and the NHI beneficiaries were assigned personal identification numbers that enabled us to trace the study patients throughout the follow-up period. Patients with lung cancer can apply for a catastrophic illness card, scrutinized by 2 pulmonologists according to NHIA guidelines. Patients with catastrophic illness card can be exempted from paying a copayment for further advanced therapy in Taiwan. However, certain limitations in our study should be considered. First, Details of pathological staging results and Eastern Cooperative Oncology Group performance status for the study patients were not available in the LHID-CIP data. Second, the study was merely observational and treatment allocation based on physician-specific practice, which was not randomized, thus having a low strength level.

## Conclusions

The use of IS for patients with lung cancers undergoing surgical resection via VATS is beneficial in lowering hospitalization costs and decreasing the incidence of pneumonia occurrence. The chest surgeon may recommend the use of IS to patients with lung cancers undergoing VATS. However, prospective randomized trials are warranted to confirm the effectiveness of IS following thoracic surgery in the future.

## Data Availability

The data and materials are not available for public use because the National Health Insurance Research Database belongs to the National Health Insurance Administration (NHIA). All the related data have been provided in the manuscript.

## Abbreviations

COPD: Chronic obstructive pulmonary disease
ED: Emergency department
ICD-9-CM: International Classification of Diseases, Ninth Revision, Clinical Modification
IS: Incentive spirometry
LHID-CIP: Longitudinal Health Insurance Database for Catastrophic Illness Patients
NHI: National Health Insurance
NHIA: National Health Insurance Administration
NHIRD: National Health Insurance Research Database
NSCLC: Non-small cell lung cancers
VATS: Video-assisted thoracic surgery
VC: Vital capacity
